# Estimating the expected planting area of double- and single-season rice in the Hunan-Jiangxi region of China by 2030

**DOI:** 10.1038/s41598-022-10357-y

**Published:** 2022-04-13

**Authors:** Min Huang, Jiana Chen, Fangbo Cao

**Affiliations:** Rice and Product Ecophysiology, Key Laboratory of Ministry of Education for Crop Physiology and Molecular Biology, Hunan Agricultural University, Changsha, 410128 China

**Keywords:** Socioeconomic scenarios, Sustainability, Biogeography

## Abstract

The development of double-season rice cropping systems has made a considerable contribution toward achieving rice self-sufficiency in China. However, the planting area for double-season rice has sharply decreased in the Hunan-Jiangxi region (the most important producing region of double-season rice in China) as a result of the conversion from double- to single-season rice cropping systems (referred as the rice “double-to-single” phenomenon). Due to concerns about the negative effect of the “double-to-single” phenomenon on rice self-sufficiency in China, we have estimated the planting area of double- and single-season rice in the Hunan-Jiangxi region that will be needed by 2030 to maintain the contribution to China’s rice production, based on the most recent 10 years (2011–2020) of historical data available. The results of our analysis can provide guidance for the government’s decision-making when planning the planting area of double- and single-season rice in the Hunan-Jiangxi region.

## Introduction

China is the world’s most populous country, with a population of over 1.4 billion people, or 18% of the world human population^1^. However, China has only about 9% of the 1.4 billion hectares of total arable land in the world^2^. The question of “who will feed China?” raised by Dr. Lester R. Brown in 1995 is still worthy of consideration today, and ensuring food security remains a top priority for the Chinese government^3^.

Rice is the staple food on dining-tables of over 65% of the population in China; thus, adequate rice production is critical to ensure food security in China^4^. In order to produce more rice on the limited amount of arable land available, double-season rice cropping systems, which involve successively growing early-season rice (ESR) and late-season rice (LSR) from March to November within a single calendar year, have been extensively developed in southern China^5^. The development of double-season rice cropping systems has made a considerable contribution toward achieving rice self-sufficiency in China^6^.

**Figure 1:**
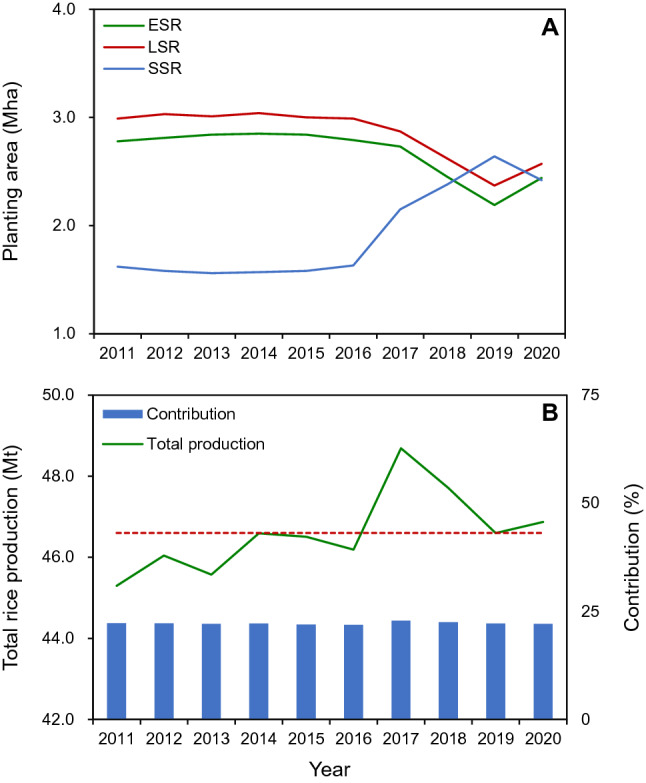
(**A**) Planting areas (million hectares, Mha) for early-season rice (ESR), late-season rice (LSR), and single-season rice (SSR) in the Hunan-Jiangxi region and (**B**) total rice production (million tons, Mt) in the Hunan-Jiangxi region and the contribution of the Hunan-Jiangxi region to total rice production in China from 2011 to 2020. In (**B**), the dashed line represents the average rice production during 2011–2020. The rice planting area and total rice production in the Hunan-Jiangxi region were calculated based on data collected from the Hunan Provincial Bureau of Statistics^8^ and the Jiangxi Provincial Bureau of Statistics^9^. The contribution of the Hunan-Jiangxi region to rice production in China is the percentage of total rice production in the Hunan-Jiangxi region to the total rice production in China. Data for total rice production in China were collected from the National Bureau of Statistics of China^10^.

Hunan and Jiangxi are the top two double-season rice producing provinces in China^7^. However, in recent years, the planting area devoted to double-season rice has sharply decreased in the Hunan-Jiangxi region as a result of the conversion from double- to single-season rice (SSR) cropping systems (referred as the rice “double-to-single” phenomenon) (Fig. 1A). A reduced rural labor supply and rising labor wages due to urbanization and economic growth are the key driving forces for the rice “double-to-single” phenomenon^11^. Fortunately, the rice “double-to-single” phenomenon has not resulted in a decrease in total rice production in the Hunan-Jiangxi region (Fig. 1B). During the most recent 10 years (2011–2020), the total rice production in the Hunan-Jiangxi region has been ranged from 45.3 to 48.7 million tons (Mt) with an average of 46.6 Mt, and the contribution of the Hunan-Jiangxi region to rice production in China has been maintained at ~ 22%.

Because China’s population is still growing^12^, China must continue to increase rice production. The domestic demand for rice grain in China is expected to reach 217 Mt by 2030, when the population of China is expected to stabilize^6^. To meet this demand, the Hunan-Jiangxi region will need to produce 47.7 Mt of rice grains, assuming that the contribution of the Hunan-Jiangxi region to rice production in China remains at the level of the most recent 10 years (~ 22%) (Fig. 1B). This expected rice production (ERP) is 1.1 Mt higher than the average total rice production during the most recent 10 years. In order to avoid the negative effect of the “double-to-single” phenomenon on achieving the ERP in the Hunan-Jiangxi region by 2030, it is necessary to estimate how much planting area of double-season rice will be needed in this region by this point in time.

**Figure 2:**
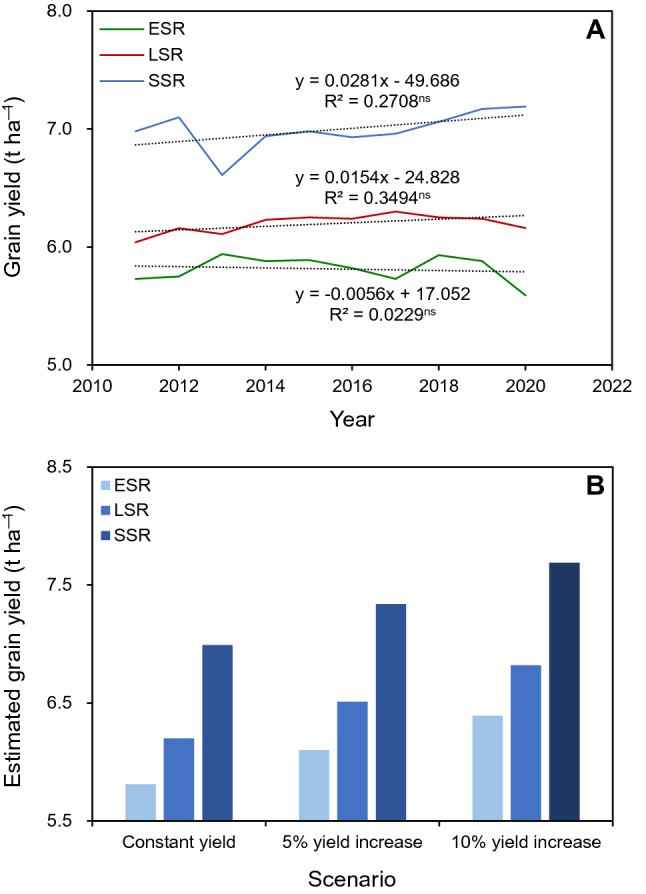
(**A**) Grain yields from 2011 to 2020 and (**B**) estimated grain yields by 2030 under three scenarios for early-season rice (ESR), late-season rice (LSR), and single-season rice (SSR) in the Hunan-Jiangxi region. The grain yields from 2011 to 2020 were calculated based on data collected from the Hunan Provincial Bureau of Statistics^8^ and the Jiangxi Provincial Bureau of Statistics^9^. In (**A**), ns denotes non-significant trend at the 0.05 probability level (Statistix 8.0, Analytical software, Tallahassee, FL, USA). In (**B**), the baseline yield for all three scenarios is the average grain yields during 2011–2020.

**Figure 3:**
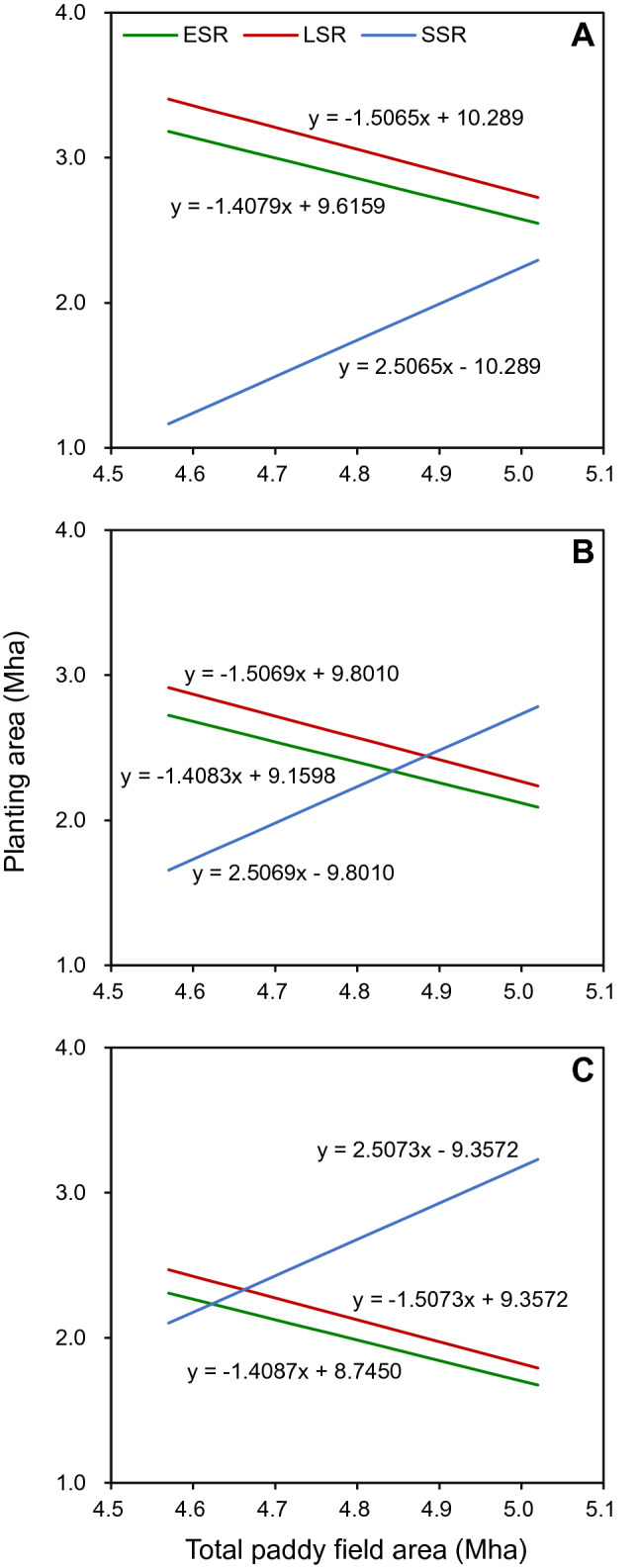
Estimated planting areas for early-season rice (ESR), late-season rice (LSR), and single-season rice (SSR) in the Hunan-Jiangxi region that will be required to achieve the expected rice production by 2030 under three scenarios: (**A**) constant yield scenario, (**B**) 5% yield increase scenario, and (**C**) 10% yield increase scenario. Mha is million hectares.

The ERP can be expressed by the following formula: ERP = EPA_ESR_ × EGY_ESR_ + EPA_LSR_ × EGY_LSR_ + EPA_SSR_ × EGY_SSR_, where EGY_ESR_, EGY_LSR_, and EGY_SSR_ are the estimated grain yields of ESR, LSR, and SSR, respectively; and EPA_ESR_, EPA_LSR_, and EPA_SSR_ are the estimated planting areas for ESR, LSR, and SSR, respectively. We assume the following conditions in the Hunan-Jiangxi region by 2030: (1) the total paddy field area will be maintained in the range of 4.57–5.02 million hectares (Mha) that was planted during the years 2011–2020^8,9^; (2) the ratio of EPA_LSR_ to EPA_ESR_ is the same as the average ratio of planting area of LSR to ESR during 2011–2020 (i.e., 1.07) (Fig. 1A); (3) EPA_SSR_ is the difference between the total paddy field area and the EPA_LSR_; and (4) EGY_ESR_, EGY_LSR_, and EGY_SSR_ are projected under three scenarios: (1) constant yield scenario, (2) 5% yield increase scenario, and (3) 10% yield increase scenario (Fig. 2). The baseline yield for all three scenarios is the average grain yields during 2011–2020. The EPA_ESR_, EPA_LSR_, and EPA_SSR_ in the Hunan-Jiangxi region needed to achieve the expected rice production by 2030 were obtained by solving the above formula and are shown in Fig. 3.

The results presented in Fig. 3 provide guidance and models for the government’s decision-making process in the planning planting areas for ESR, LSR, and SSR in the Hunan-Jiangxi region. In brief, farmers will need to plant 2.55–3.18 Mha of ESR, 2.73–3.40 Mha of LSR, and 1.17–2.29 Mha of SSR under the constant yield scenario, 2.09–2.72 Mha of ESR, 2.24–2.91 Mha of LSR, and 1.66–2.78 Mha of SSR under the 5% yield increase scenario, and 1.67–2.31 Mha of ESR, 1.79–2.47 Mha of LSR, and 2.10–3.23 Mha of SSR under the 10% yield increase scenario in the Hunan-Jiangxi region by 2030 depending on the total available paddy field area.

One thing to note here is that the actual planting areas for ESR (2.44 Mha) and LSR (2.57 Mha) in 2020 are below the estimated lower limits of planting areas for ESR (2.55 Mha) and LSR (2.73 Mha) that will be needed by 2030 under the constant yield scenario, while the actual planting area for SSR in 2020 (2.42 Mha) is above the estimated upper limit for SSR (2.29 Mha) that will be needed by 2030 under the constant yield scenario (Figs. 1A and 3A). This finding indicates that it is urgent to avoid a further aggravated “double-to-single” phenomenon while maintaining the total paddy field area in the Hunan-Jiangxi region. Because it is not an easy task to maintain the total paddy field area under the projected scenario for urban expansion^13^, the government should prepare an alternative to reverse the “double-to-single” phenomenon in the Hunan-Jiangxi region. Increasing the mechanized level of farming operation and improving economic returns to farmers are two key aspects for the government to take into account to promote the development of double-season rice.

Although the current planting area of double-season rice can fully meet the requirement for achieving the ERP in the Hunan-Jiangxi region by 2030 under both the 5% and 10% yield increase scenarios, there is some difficulty in reaching the yield increase targets. In recent years, the planting area of high-quality rice varieties has been rapidly increased in China^14^. However, grain yield is generally not very high for high-quality rice varieties, although no genetic linkage has been identified between grain yield and quality in rice^15^. Hence, great efforts are required to develop rice varieties with both high quality and high yield. In addition, rice yields are determined not only by the variety but also by environments and crop management practices. Soil nutrient deficiencies, unfavorable climatic conditions (e.g., heat, cold, and drought), and pest infestations have always been major yield-limiting factors for rice production in China^16^. Therefore, great efforts are also required to: (1) improve soil fertility of low- and medium-yielding rice fields and optimize nutrient management practices; (2) develop climate-smart agriculture practices for alleviating climatic stresses; and (3) promote integrated pest management practices.

## Data Availability

All data generated or analysed during this work are included in the article.
